# Evaluation of Omnigene-Sputum for Preservation of Sputum Samples for Diagnosis of *Mycobacterium tuberculosis*

**DOI:** 10.3390/tropicalmed8070367

**Published:** 2023-07-17

**Authors:** Edson Mambuque, Belén Saavedra, Barbara Molina-Moya, Dinis Nguenha, Esther García-García, Silvia Blanco, Neide Gomes, Joanna Ehrlich, Helder Bulo, Shilzia Munguambe, Helio Chiconela, Sozinho Acacio, José Domínguez, Alberto L. García-Basteiro

**Affiliations:** 1 Centro de Investigação em Saúde de Manhiça (CISM), Maputo 1929, Mozambique; 2 ISGlobal, Hospital Clínic, Universitat de Barcelona, 08026 Barcelona, Spain; 3 Institut d’Investigació Germans Trias i Pujol, Universitat Autònoma de Barcelona, 08916 Badalona, Spain; 4 Centro de Investigación Biomédica en Red de Enfermedades Respiratorias (CIBERES), 08916 Badalona, Spain; 5 National Tuberculosis Control Program (PNCT), Maputo 1929, Mozambique; 6 Centro de Investigación Biomédica en Red de Enfermedades Infecciosas (CIBERINFEC), 08026 Barcelona, Spain

**Keywords:** omnigene, sample transportation, tuberculosis

## Abstract

In several low-income countries, the transport of sputa could take up to one week to reach the laboratories, resulting in increased contamination rates and a loss of growth. The aim of this study was to evaluate the effect of the OMNIgene-SPUTUM in preserving *Mycobacterium tuberculosis* on sputum samples simulating three hypothetical scenarios for conservation and/or decontamination: (1) sputum was mixed with OMN and conserved at room temperature for five days and then processed for culture (OMN); (2) sputum cultures followed the routine standing operating procedure at day 0 (STD); and (3) sputum samples were kept at room temperature for five days and mixed with the standard decontamination reagent (SDT5) and then processed for culture. The positivity rate based on smear microscopy was 36.4%, 29.1%, and 27.3% for STD, STD5, and OMN, respectively. The proportion of positive results by liquid culture (MGIT) was 39.1% (43/110) for STD, 26.4% (29/110) for STD5, and 20.0% for OMN (22/110). The overall concordance of liquid culture results was 51.8% (57/110): 37.3% (41/110) for negative results, 11.8% (13/110) for MTBC growth, and 2.7% (3/110) for contaminated results. The OMN arm showed better performance in solid culture than in liquid culture, with a notable reduction in contaminated results.

## 1. Introduction

Tuberculosis (TB) remains one of the top 10 major public health problems worldwide, with an estimated 10.6 million people falling ill from TB annually. TB caused 1.4 million deaths among HIV-negative people and an additional 187,000 deaths among people living with HIV (PLHIV) in 2021 [1]. Accurate, sensitive, and high-quality diagnostic testing, usually performed in centralized laboratories in urban centers, is crucial to improving TB diagnosis and identifying drug resistance. If specimens cannot reach centralized laboratories under conditions that preserve specimen integrity, the performance of downstream tests and their potential impact will be undermined [2].

In 2015, Mozambique responded to the current WHO framework for universal DST and established six reference laboratories to perform cultures, 85 sites to perform Xpert^®^ MTB/RIF, and 400 smear microscopy laboratories, reaching a ratio of 1 culture laboratory per 5 million people in an area of 26,753 km [3,4,5]. Nevertheless, the conservation and transport of sputa to those laboratories often encounter operational hurdles. The Global Laboratory Initiative (GLI) recommends the use of cold chains for long-distance transport and the initiation of cultures within 48 h of collection [2]. Sample shipment to central laboratories requires reliable and continuous access to refrigeration in order to maintain sample integrity. Failures in this chain will result in higher contamination rates and the loss of available mycobacteria, which will imply re-sampling and delays in initiating effective treatment [6,7].

Novel pre-analytical technologies may address these issues, although they need to be flexible to assist laboratories and national programs most widely and effectively [8]. OMNIgene-SPUTUM (DNA Genotek, Ottawa, ON, Canada; hereinafter OMN) is a non-toxic and highly stable reagent that can be applied to sputum samples prior to testing to liquify and decontaminate fresh or frozen sputum samples to preserve the viability of *Mycobacterium tuberculosis* during transportation [5,9]. The liquid-based reagent enables shipping temperatures from 4 °C to 40 °C and storage of sputum for up to 8 days. Furthermore, samples treated with OMN are compatible with smear microscopy, solid and liquid cultures, and molecular assays [9]. When OMN is added, the cold chain is not required for shipping, and samples are directly compatible with molecular assays and gold standard TB tests, such as smear microscopy, solid and liquid culture, and the Cepheid Xpert MTB/RIF assay (Cepheid, Sunnyvale, CA, USA) [10].

Previous studies conducted in Africa using OMN have not consistently shown its ability to reduce contamination or increase the yield from cultures compared to the conventional method. However, some studies demonstrated a decrease in contamination rates compared to Lowenstein–Jensen media (LJ) and suppression of contaminants [3,10,11,12]. This study aimed to evaluate the effect of OMN on the recovery of *M. tuberculosis* by comparing different conservation and decontamination methods in a high-TB and HIV-burden setting in Southern Mozambique.

## 2. Materials and Methods

### 2.1. Study Design

This is a prospective evaluation of the OMN reagent on the recovery of viable *M. tuberculosis* complexes in sputum samples from presumptive TB patients. We compared the agreement of culture results (liquid and solid) by using a control arm (standard procedures for culturing) and two conservation and decontamination strategies aiming to simulate 5-day delay scenarios in sample transport and laboratory processing. The study had three arms. Arm 1 (standard, STD): samples followed the reference standard method for culture on the day they were received. Arm 2 (OMN): samples were mixed with OMN and kept at room temperature for five days. Arm 3 (standard day 5, STD5): samples were kept at room temperature for 5 days and mixed with the standard decontamination reagent.

### 2.2. Study Setting and Population

The present study was conducted by the Centro de Investigação em Saúde de Manhiça (CISM) in the district of Manhiça, a rural area 80 km away from the capital city of Maputo in Southern Mozambique. This is a high TB and HIV burden region, with an estimated incidence rate for TB in Mozambique of 361 per 100,000 population, with 34% of the TB-notified cases being HIV positive [13]. The prevalence of HIV infection in adults is around 35% [14,15], and the case notification rate in the district is around 500 per 100,000 people [16], although it is much higher in PLHIV [17].

Consecutive adults with presumptive TB were identified at the Manhiça District Hospital (HDM) using the WHO-recommended symptom screening algorithm, in which the presence of cough, fever, night sweats, or unintentional weight loss was assessed. Patients who screened positive for TB-compatible symptoms were offered the opportunity to participate in the study. The clinical data of the patients was collected from their clinical histories. Informed consent was obtained from all subjects involved in the study.

On the other hand, the experimental data was provided by the CISM, which was where the samples were processed and the cultures performed.

### 2.3. Study Procedures

**Figure 1:**
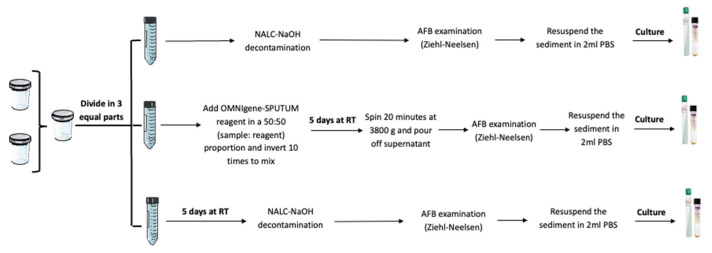
Summary of study arms.

Two sputum samples per patient were collected in sterile tubes (Figure 1) and sent to the CISM Laboratory. Both samples were transferred to a sterile screw-capped tube and mixed through vortexing. Afterward, the mixture was split into three equal volumes in conical tubes of 50 mL. Each sample was processed according to study procedures (Arm STD, OMN, or STD5). Figure 1 shows the detailed procedures for each arm.

Standard decontamination procedure (STD): Samples were digested and decontaminated using the Kubica method (2% NALC-NaOH). Briefly, the sample was mixed with an equal volume of NaOH-N-Acetyl-L-Cysteine-sodium citrate solution and treated for 20 min, vortexing every 5 min. After that, a phosphate buffer (PBS) (pH 6.8) was added to establish a total volume of 45 mL. Samples were centrifuged at 3000× *g* for 20 min, the supernatant was poured off. After decontamination, 1–2 mL of PBS (pH 6.8) was added to reconstitute the sediment, which was used for smear microscopy and solid and liquid cultures. This process was used in Arm 1 (STD, on the same day the samples arrived at the laboratory) and in Arm 3 (STD5, after 5 days at room temperature).

OMN procedure (arm 2): Sputum samples were processed according to the manufacturer’s instructions. Briefly, the sample was mixed with an equal volume of OMN reagent, kept at room temperature for 5 days, and centrifuged at 3800× *g* for 20 min. Then, the supernatant was poured off, and the sediment was prepared for smear microscopy and solid and liquid culture.

Ziehl–Neelsen staining: All sediments were smeared using the Ziehl–Neelsen (ZN) method. They were stained with carbol fucsin for 10 min, decolorized with acid-alcohol for 2 min, and counter-stained with methylene blue for 4 min. Smears were analyzed under optical microscopy at 100-fold magnification. Smears were graded as scanty (1 to 9 bacilli per 100 fields), 1 + (10–99 bacilli in 100 fields), 2 + (1–10 bacilli per field in 50 fields), and 3 + (>10 bacilli per field in 20 fields).

Cultures and identification of mycobacteria: After sediment reconstitution, for liquid culture, 500 microliters of the pellet were immediately inoculated into the Mycobacterial Growth Indicator Tube (Becton Dickinson MGIT) and incubated in the Bactec MGIT 960. Also, 200 microliters were inoculated into LJ for solid culture and incubated at 35–37 °C until growth was observed or was reported as negative after 42 days for MGIT culture or 8 weeks for LJ culture.

In order to differentiate growth due to *M. tuberculosis complex* (MTBC) or non-tuberculous mycobacteria (NTM), positive cultures were tested by ZN stain and immunochromatography (BD MGIT™ TB identification test, Becton Dickinson, Franklin Lakes, NJ, USA) to detect the MPT64 antigen specifically produced by the *M. tuberculosis* complex.

### 2.4. Statistical Methods

To compare the performance of OMN and STD5 protocols against the reference standard (STD arm), we assessed agreement between each method by calculating the Cohen’s kappa coefficient (k ± standard error). The k-values were assigned to a ‘‘strength of agreement” as follows: 0.01–0.20 poor; 0.21–0.40 fair; 0.41–0.60 moderate; 0.61–0.80 substantial; and 0.81–1.00 good. We used the two-sided test statistic for paired data and its exact version for small groups to test whether there was a systematic difference in sensitivity and specificity between the OMN and STD5 arms test results in comparison with the reference standard (STD). A *p*-value of 0.05 was considered the threshold for statistical significance. All analyses were conducted with SPSS statistical software for Windows (SPSS, version 14.0; SPSS Inc., Chicago, IL, USA).

## 3. Results

Between March 2016 and November 2016, a total of 110 participants with presumptive pulmonary TB were enrolled in this study and provided diagnostic sputum samples at the HDM. The mean age of participants was 40.9 [IQR22.8; 59.0], and 54.6% of them were male. Of the 110 participants included, 34 were microbiologically cultured and confirmed as having TB, and 28 accepted the treatment. From the non-microbiologically confirmed cases, 35 clinically diagnosed cases of TB received also treatment.

After processing the samples, 36.4% (40/110) were initially sputum smear positive by STD protocol, 27.3% (30/110) were positive by OMN protocol, and 29.1% (32/110) by STD5 (Table 1). The proportion of positive results (MTBC or NTM) by liquid culture (MGIT) was 39.1% (43/110) for STD, 20.0% (22/110) for OMN, and 26.4% (29/110) for STD5. The proportion of positive solid cultures (LJ) was 24.6% (27/110) for STD, 21.8% (24/110) for OMN, and 25.5% (28/110) for STD5. The proportion of MTBC recovery in liquid culture was higher in STD 30.9% (34/110) and STD5 24.5% (27/110) compared to OMN 19.1% (21/110).

### 3.1. Agreement among Techniques and Procedures Sputum Smear (Table 2)

The overall proportion of concordant results between STD, OMN, and STD5 was 75.5% (83/110), of whom 61.8% (68/110) were negative, 4.6% (5/110) graded as 3+, 1.8% (2/110) graded as 2+, 4.6% (5/110) graded as 1+, and 2.7% (3/110) graded as scanty.

Comparison of STD and OMN showed 90.9% agreement (100/110, k = 0.80 ± 0.061 classified as substantial agreement). STD and STD5 achieved the same results in 89.1% of cases (98/110, k = 0.751 ± 0.07, classified as substantial agreement). Concordance between STD5 and OMN occurred in 92.7% of patients (102/110; k = 0.865 ± 0.53, classified as good agreement).

Overall, discordant results among the 3 arms (considered discordant when a certain result does not agree in at least one of the arms) were obtained for eighteen samples (Table 2). From all of them that were positive for STD; only three were positive for OMN and also positive for STD5. Conversely, of those negative for OMN, only one was positive for STD5.

**Table 2:** Smear microscopy results obtained with the different decontamination methods for the samples with discordant results.

STD ^1^	OMN ^2^	STD5 ^3^	*n*
Positive (3+)	Positive (2+)	Negative	1
Positive (3+)	Negative	Negative	2
Positive (3+)	Positive (1+)	Positive (2+)	2
Positive (3+)	Positive (1+)	Positive (3+)	1
Positive (3+)	Negative	Positive (3+)	1
Positive (2+)	Positive (3+)	Positive (3+)	1
Positive (2+)	Positive (1+)	Positive (1+)	1
Positive (2+)	Positive (scanty)	Negative	1
Positive (2+)	Negative	Negative	2
Positive (1+)	Positive (3+)	Positive (scanty)	1
Positive (1+)	Positive (scanty)	Positive (1+)	1
Positive (1+)	Positive (scanty)	Negative	1
Positive (1+)	Negative	Negative	3

^1^ STD: Sputum cultures followed the routine standing operating procedure at day 0 OMNIgene-SPUTUM reagent. ^2^ OMN: Sputum was mixed with OMNIgene-SPUTUM reagent, conserved at room temperature for five days, and then processed for culture. ^3^ SDT5 sputum samples were kept at room temperature for five days, mixed with the standard decontamination reagent, and then processed for culture.

### 3.2. Liquid Culture (Table 3)

Overall, the proportion of concordant results among the 3 arms, STD, OMN, and STD5, was 51.8% (57/110), of which 37.3% (41/110) were negative, 11.8% (13/110) were positive for MTBC, and 2.7% (3/110) were contaminated (Table 3).

**Table 3:** Liquid culture discordant results obtained with the different decontamination methods.

STD ^1^	OMN ^2^	STD5 ^3^	*n*
Negative	Negative	Contaminated	7
Negative	MTBC ^4^	MTBC	1
MTBC	Negative	Negative	3
MTBC	MTBC	Negative	1
MTBC	MTBC	Contaminated	4
Contaminated	Negative	Contaminated	8
Contaminated	Negative	Negative	13
Contaminated	Negative	MTBC	1
Contaminated	MTBC	Contaminated	2

^1^ STD: Sputum cultures followed the routine standing operating procedure at day 0 OMNIgene-SPUTUM reagent. ^2^ OMN: Sputum was mixed with OMNIgene-SPUTUM reagent, conserved at room temperature for five days, and then processed for culture. ^3^ SDT5 sputum samples were kept at room temperature for five days, mixed with the standard decontamination reagent, and then processed for culture. ^4^ MTBC: *Mycobacterium tuberculosis* complex.

Comparison between arms showed an agreement of 61.8% (68/110) between STD and OMN (k = 0.35 ± 0.15, ‘Fair’ strength of agreement) and 70.9% (78/110) between STD and STD5 (k = 0.541 ± 0.13, ‘Moderate’ strength of agreement). The agreement between OMN and STD5 was 67.2% (74/110) (k = 0.38 ± 0.16 ‘Fair’ strength of agreement).

The sensitivity and specificity observed in OMN were 0.71 (0.42–0.92) and 0.97 (0.86–1), respectively, with reference to STD, and 0.83 (0.52–0.98) and 0.97 (0.87–1), respectively, with reference to STD5, with a *p*-value > 0.05.

When comparing STD and OMN arms, 25.4% of samples showed discordant results (28/110). Of them, three MTBC-positive cultures by OMN were contaminated with or negative for STDs. The opposite occurred in the other three cases, in which STD preserved MTBC whereas OMN cultures were negative. When comparing STD and STD5, the proportion of discordant results was slightly higher at 27.2% (30/110); 8 MTBC were not recovered after 5 days of incubation, whereas 2 MTBC cases were recovered in the STD5 arm that were negative or contaminated at baseline (STD).

### 3.3. Solid Culture (Lowenstein Jensen)

Testing of solid culture (Table 4) through LJ medium resulted in 50% (55/110) concordant results across the three arms (STD, OMN, and STD5), of which 36.4% (40/110) were negative, 9.1% (10/110) were MTBC, and 4.5% (5/110) were contaminated.

Comparison among arms showed the agreement between STD and OMN to be 61.5% (68/110 k = 0.40 ± 0.15, the strength of agreement is considered to be ‘Fair’), while the agreement between STD5 and OMN was 67.3% (74/110 k = 0.42 ± 0.14, the strength of agreement is considered to be ‘Moderate’).

The sensitivity and specificity observed in OMN were 0.73 (0.39–0.94) and 0.92 (0.80–0.98) with reference to STD, respectively, and 0.90 (0.55–1) and 0.95 (0.83–0.99), respectively, with reference to STD5 with a *p*-value > 0.05.

The number of discordant results between the three arms was higher for solid cultures. Between STD and OMN cases, 35.4% of results differed (39/110). From the 15 MTBC-positive samples in the STD arm, 8 were negative and 2 were contaminated in the OMN arm. Conversely, OMN recovered six samples from contaminated samples by the standard method.

For standard comparisons, a similar proportion of results differed (38/110). Twelve cases of MTBC were lost after 5 days of delay in culture, although 7 samples that were contaminated at baseline were positive in the STD5 arm.

## 4. Discussion

The present study aimed to evaluate the performance of the sample preservative OMN in a scenario where there is a 5-day delay before processing the sample for culture methods. This method was compared to two other strategies that involved standard decontamination procedures: (a) routine same-day decontamination and processing (STD) and (b) delaying five days before starting routine decontamination and processing procedures (STD5). Our findings show that the overall proportion of MTBC positive results by STD in liquid culture (MGIT) was higher (30.1%) than was observed in OMN (19.1%) and in STD5 (24.5%). OMN demonstrated a poor overall recovery of MTBC, which was slightly improved in solid medium.

There are other studies evaluating the performance of OMN in different settings that use different reference methods and microbiological tests [2,3,10,12,18,19,20,21,22]. Azam et al. described a good recovery of MTBC by using OMN with a high positive rate in LJ (10% in the same-day and 56% in the 5-day arms) and a low positive rate in MGIT (52% and 28% lower in the same-day and 5-day arms, respectively), but no superiority of OMN when using molecular bacterial load assay to count viable MTBC cells [3]. Maharjan et al. found that the overall average time to LJ culture positivity was not significantly affected between OMN and STD after transport without refrigeration [6]. Kelly–Cirino et al. reported that the recovery with OMN-treated samples required, on average, 5.6 additional days to become MGIT-positive [10,19]. The low proportion of positive cultures across all media may be associated with the alkaline pH following re-suspension. It is generally well understood that high pH mediums can cause injury or death to mycobacteria and delay mycobacterial revival [12]. Bimba et al. evaluated OMN for the preservation of sputum for Xpert MTB/RIF testing in Nigeria. They reported that OMN-stored and fresh sputum samples had similar Xpert results [23]. The development of independent studies in different settings, although performing in some way similar designs, is very needed for increasing the evidence and for facilitating robust decisions about implementation supported by a higher number of studies and data. The three arms and the processing timing included in our design (OMN, STD, and STD5) capture real scenarios in low-resource settings, thus allowing fair comparisons between them.

However, OMN was associated with a lower number of contaminated culture results when compared to other methods (Table 1). The highest reduction in contaminated culture results was detected with MGIT culture (4.5% on OMN, 16.4% on STD, and 20.9% on 5STD). Other studies [10,20,21,24] have shown OMN to reduce culture contamination from 12% to 2% [21] and 10% to 3% [10] for LJ and from 13% to 0.3% [20] and 5% to 2% [10] for MGIT. Decreasing the rate of contamination results is especially important in low-resource settings with a high TB burden, where contamination of the samples may be more frequent. Probably, to reduce contamination, OMN should be used immediately after the samples are collected and for samples that should be sent from long distances and that will take several times to arrive at the laboratory. However, this lower contamination rate should not come at the expense of a lower positivity rate. This was observed in our study, where the contamination and positivity rates of cultures were notably different between STD and OMN. Zallet et al. suggest the OMN reagent might interfere with some components of the MGIT or directly affect mycobacterial growth, causing both poor recovery and delayed time to detection [12].

OMN showed a higher proportion of positivity when compared to STD5, but a lower proportion when compared to STD. The impact of OMN on smear microscopy in other studies was variable, in some cases yielding lower smear grading when compared to STD and/or STD5 [6,21]. By contrast, other studies show a similar proportion of smear-positive results using OMN and routine NALC-NaOH on the same day of collection, but also when performed after several days of transport with and without refrigeration [9,10].

### Limitations

This study has some limitations. First, the sample size was somewhat small to be able to draw more meaningful conclusions. Second, small variabilities in the quantity of the bacterial load of specimens that were put in each arm may play a role in the observed results. Third, despite similar training, there might be some variability in the application of the decontamination protocols by the two laboratory technologists involved in the study. To reduce the risk of high variability, the laboratory participants received intensive training on the technology; both are experienced TB laboratory technologists, and they were supervised and monitored throughout the study. The fact that only two technologists from only one laboratory were involved significantly reduced the bias in the performance of the techniques and therefore increased the reliability of the results. Lastly, time to positivity for both culture methods, not recorded in this study, could have provided some relevant information to support the lower viable bacillary load in the OMN arm. On the other hand, the comparison of the impact of the positive rate of molecular methods (i.e., Xpert MTB/RIF) would have been interesting. However, due to the fact that the OMN was specifically designed for preserving *M. tuberculosis* viability at ambient temperature while liquefying and decontaminating sputum samples, it was not considered in the study protocol.

## 5. Conclusions

In summary, the use of the OMN reagent decreased contamination rates in both liquid and solid cultures. But the study shows that OMN was associated with a lower proportion of positive results in MGIT and a similar proportion with LJ when compared to the standard NALC-NaOH decontamination methods. The lower yield of positive liquid culture results would not support its use in our setting.

## Figures and Tables

**Table 1:** Summary of observed results obtained with the different decontamination methods.

Test	Result	STD ^1^ % (*n*)	STD5 ^2^ % (*n*)	OMN ^3^ % (*n*)
Smear	Negative	63.6 (70/110)	72.7 (80/110)	70.9 (78/110)
	Scanty	3.6 (4/110)	4.5 (5/110)	6.4 (7/110)
	1+	10.0 (11/110)	6.4 (7/110)	8.2 (9/110)
	2+	6.4 (7/110)	9.1 (10/110)	8.2 (9/110)
	3+	16.4 (18/110)	7.3 (8/110)	6.4 (7/110)
MGIT	Negative	44.6 (49/110)	52.7 (58/110)	75.5 (83/110)
	MTBC ^4^	30.9 (34/110)	24.5 (27/110)	19.1 (21/110)
	NTM ^5^	8.2 (9/110)	1.8 (2/110)	0.9 (1/110)
	Contaminated	16.4 (18/110)	20.9 (23/110)	4.5 (5/110)
	Rescued by OMN ^6^	3.9 (3/76)	8.4 (7/83)	
LJ ^7^	Negative	52.7 (58/110)	48.2 (53/110)	65.5 (72/110)
	MTBC	21.8 (24/110)	20.9 (23/110)	21.8 (24/110)
	NTM	2.7 (3/110)	2.7 (5/110)	0
	Contaminated	22.7 (25/110)	26.4 (29/110)	12.7 (14/110)
	Rescued by OMN	7.0 (6/86)	9.2 (8/87)	

^1^ STD: Sputum cultures followed the routine standing operating procedure at day 0 OMNIgene-SPUTUM reagent. ^2^ SDT5 sputum samples were kept at room temperature for 5 days, mixed with the standard decontamination reagent, and then processed for culture. ^3^ OMN: Sputum was mixed with OMNIgene-SPUTUM reagent, conserved at room temperature for five days, and then processed for culture. ^4^ MTBC: *Mycobacterium tuberculosis* complex. ^5^ NTM—Non-Tuberculosis Mycobacteria. ^6^ Rescued by OMN: were estimates based on the results from OMN that were MTBC but discrepant either from STD or STD5, and results that were considered negative, contaminated, or NTM. LJ ^7^. Löwenstein–Jensen, solid culture media.

**Table 4:** Solid culture discordant results obtained with the different decontamination methods.

STD ^1^	OMN ^2^	STD5 ^3^	*n*
Negative	Negative	Contaminated	10
Negative	Contaminated	Contaminated	3
Negative	MTBC ^4^	MTBC	3
MTBC	Negative	Negative	3
MTBC	Negative	MTBC	1
MTBC	Negative	Contaminated	3
MTBC	Negative	Contaminated	1
MTBC	MTBC	Negative	2
MTBC	MTBC	Contaminated	3
MTBC	Contaminated	MTBC	2
Contaminated	Negative	Negative	4
Contaminated	Negative	MTBC	3
Contaminated	Negative	Contaminated	8
Contaminated	MTBC	Negative	1
Contaminated	MTBC	MTBC	3
Contaminated	MTBC	Contaminated	2
Contaminated	Contaminated	Negative	1
Contaminated	Contaminated	MTBC	1

^1^ STD: Sputum cultures followed the routine standing operating procedure at day 0 OMNIgene-SPUTUM reagent. ^2^ OMN: Sputum was mixed with OMNIgene-SPUTUM reagent, conserved at room temperature for five days, and then processed for culture. ^3^ SDT5 sputum samples were kept at room temperature for five days, mixed with the standard decontamination reagent, and then processed for culture. ^4^ MTBC: *Mycobacterium tuberculosis* complex.

## Data Availability

The datasets generated during and/or analyzed during the current study are not publicly available, but are available from the corresponding author on reasonable request.
